# A Study of the Demographics of Web-Based Health-Related Social Media Users

**DOI:** 10.2196/jmir.4308

**Published:** 2015-08-06

**Authors:** Shouq A Sadah, Moloud Shahbazi, Matthew T Wiley, Vagelis Hristidis

**Affiliations:** ^1^ Department of Computer Science and Engineering University of California, Riverside Riverside, CA United States; ^2^ SmartDocFinder LLC Riverside, CA United States

**Keywords:** online social media, demographics, health forums, health care disparity, drug reviews

## Abstract

**Background:**

The rapid spread of Web-based social media in recent years has impacted how patients share health-related information. However, little work has studied the demographics of these users.

**Objective:**

Our aim was to study the demographics of users who participate in health-related Web-based social outlets to identify possible links to health care disparities.

**Methods:**

We analyze and compare three different types of health-related social outlets: (1) general Web-based social networks, Twitter and Google+, (2) drug review websites, and (3) health Web forums. We focus on the following demographic attributes: age, gender, ethnicity, location, and writing level. We build and evaluate domain-specific classifiers to infer missing data where possible. The estimated demographic statistics are compared against various baselines, such as Internet and social networks usage of the population.

**Results:**

We found that (1) drug review websites and health Web forums are dominated by female users, (2) the participants of health-related social outlets are generally older with the exception of the 65+ years bracket, (3) blacks are underrepresented in health-related social networks, (4) users in areas with better access to health care participate more in Web-based health-related social outlets, and (5) the writing level of users in health-related social outlets is significantly lower than the reading level of the population.

**Conclusions:**

We identified interesting and actionable disparities in the participation of various demographic groups to various types of health-related social outlets. These disparities are significantly distinct from the disparities in Internet usage or general social outlets participation.

## Introduction

### Background

Social media have been employed in many industries to engage consumers. The health care industry has moved at a slower pace in incorporating social media because of inherent risks such as patient privacy, but recently this rate has increased to fulfill the consumers’ needs [1]. Moreover, some companies use social media to provide their employees with wellness videos in order to cut their health care costs [2].

At the same time, health care disparity is a well-studied problem in which two population groups receive unequal services [3]. This problem has been analyzed across various dimensions relating to social determinants in health, including education and income, environmental hazards, and health outcomes such as mortality, morbidity, and behavioral risk factors [4]. However, health care disparity has not been studied in terms of social media participation. This is important as Internet access and participation in health communities has the potential to improve health outcomes [5]. Hence, understanding the demographics of social outlets, which is the focus of this paper, may shed light on another facet of health care disparity.

To cover different types of Web-based social outlets, we collected data from three types of sources: (1) general Web-based social networks, namely Google+ and Twitter, (2) drug review websites, and (3) health Web forums. We measure the following demographic attributes: age, gender, ethnicity, location, and writing level. Unfortunately, much of this information is unavailable for some, or all, of the sources. For that, we built and evaluated three classifiers for gender, ethnicity, and writing level. User names were used for the gender and ethnicity classifiers. Writing level for users was calculated using modified reading level formula to ignore very long incomprehensible sentences. To extract the location of a post, we use a geocoding application programming interface (API).

### Related Work

#### Analysis of Health-Related Social Outlets

Many researchers have explored the effectiveness of Web-based social media in changing and improving the communication between providers and patients. According to Kane et al [6], 60 million Americans are using Health 2.0 applications, that is, social networks focused specifically on health care. Further, approximately 40% of Americans find an opinion in social media is more trustworthy if it conflicts with a professional’s opinion or diagnosis. Hackworth and Kunz [7] found that 80% of American adults have looked online for health-related topics. Recently, there is increased interest in analyzing the health-related content of social media [1]. Denecke and Nejdl [8] analyzed medical concepts mentioned in medical social media posts from different sources to differentiate between informative and affective posts. They found that patients and nurses tend to share personal experiences, while physicians share health-related information. Lu et al [9] studied the content of three disease-specific health communities and their relationship to five informative topics: symptoms, complications, examination, drugs, and procedures. For example, users with breast cancer are more likely to discuss examination, while users with lung cancer are more likely to discuss symptoms. Wiley et al [10] analyzed the content of Web-based social media related to pharmaceutical drugs across several dimensions, including frequently mentioned diseases, keywords, and sentiment. While the aforementioned work examined health-related social media and content, none of them studied the demographics of the participating users, which is studied in this work.

#### Measuring and Estimating Demographics of Users of Social Outlets

##### Survey-Based Methods

In 2012, a Pew Internet Research study showed that women, age 30-49, are more likely to participate in social media websites, where 75% of users are white [11]. eMarketer found that 68.9% of Hispanics use social media compared to 66.2% of the total population. Further, they showed that Hispanics are more likely to compare products online while shopping and write reviews on products [12]. However, no research has focused on health-related social media.

##### Classifier-Based Methods

Mislove et al [13] built methods to estimate both gender and ethnicity for Twitter users using the 1000 most popular first names reported by the US Social Security Administration and frequently occurring surnames reported by the 2000 US census. Gender and ethnicity methods used the reported first name and last name respectively. Mandel et al [14] analyzed the tweets related to Hurricane Irene using Mislove’s gender classifier. We build on Mislove’s work when creating our classifiers. While we also classify gender using first names, we extended these methods to screen names when a first name is not present. A related work for estimating reading levels of the US population [15] was presented to discuss limitations of low literacy patients. We measured the writing level based on this work since we did not encounter any similar work.

## Methods

### Datasets

**Figure 1:**
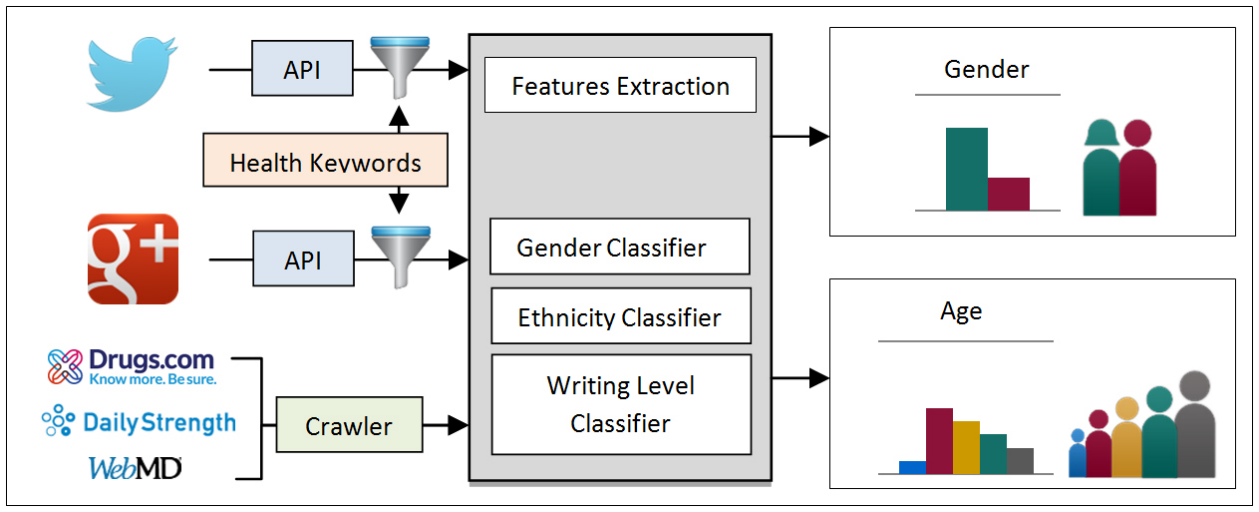
Overview of the data collection and analysis process.

Our analysis used data collected from three different types of health-related social outlets: general social networks, drug review websites, and health Web forums (Table 1 [16-23]). Google+ and Twitter were chosen as general social networks based on their popularity and number of users (we do not study Facebook because it offers no public interfaces to access its data). For drug review websites and health Web forums, three websites were selected for each, where we considered their breadth of topics and popularity. Figure 1 shows the overall process of our analysis, and Table 1 shows key statistics of each source including number of users, number of posts, and average sentence length. More information about the sources including start and end date is available in Multimedia Appendix 1.

**Table 1:** Total number of users, posts, and average sentences length for each source.

Dataset	Users, n	Posts, n	Average sentence length (in words)
TwitterHealth [16]	5,095,849	11,637,888	10.82
Google+Health [17]	86,749	186,666	9.03
Drugs.com [18]	74,461	74,461	13.85
DailyStrength/Treatments [19]	213,524	1,055,603	11.92
WebMD/Drugs [20]	122,040	122,040	13.53
Drugs.com/Answers [21]	201,126	5,948,877	6.59
DailyStrength/Forums [22]	165,045	1,128,629	13.2
WebMD [23]	155,912	320,118	15.37

Table 2 shows which of the sources provide data for each of the five demographic attributes. Two demographic attributes are not present in any source: ethnicity and writing level. We therefore created methods to automatically classify these attributes, along with gender for the sources where unavailable. *No* indicates that the demographic attribute is not provided by the source. *Yes* indicates that the demographic attribute is provided by the source. Each classifier uses a distinct part of the user profile as denoted by the table footnotes. The writing level classifier uses the Flesch-Kincaid measure based on all users’ posts [24].

**Table 2:** List of all used sources with the available attributes.

Dataset	Age	Gender	Ethnicity	Location	Writing level
TwitterHealth	No	Gender classifier^a^	Ethnicity classifier^b^	Yes	Writing level classifier
Google+Health	Yes	Yes		Yes	Writing level classifier
Drugs.com	No	Gender classifier^c^	No	No	Writing level classifier
DailyStrength/Treatments	Yes	Yes	No	Yes	Writing level classifier
WebMD/Drugs	Yes	Yes	No	No	Writing level classifier
Drugs.com/Answers	No	Gender classifier^c^	No	No	Writing level classifier
DailyStrength/Forums	Yes	Yes	No	Yes	Writing level classifier
WebMD	No	Gender classifier^c^	No	No	Writing level classifier

^a^First name.

^b^Last name.

^c^Screen name.

To filter health-related posts from Twitter and Google+, we built a list of 276 representative health-related keywords based on five categories:

1. Drugs: First we obtained a list of the 200 most popular drugs by prescriptions dispensed from RxList.com [25]. We then removed variants of the same drug (eg, different milligram dosages) resulting in 125 unique drug names. 
2. Hashtags: We selected 11 popular health-related Twitter hashtags such as #HCSM (Healthcare Communications & Social Media).
3. Disorders: We selected 81 popular disorders such as cancer and Alzheimer. 
4. Pharmaceuticals: We selected the 12 largest pharmaceutical companies such as Pfizer.
5. Insurance: We selected 44 of the biggest insurances such as Medicare and Humana. A complete list of used keywords can be found in Multimedia Appendix 1.

We used the Twitter streaming API [26], with these keywords as filters, to obtain the relevant tweets for our TwitterHealth dataset. Our Google+Health dataset was collected via the Google+ API [27], where each health-related keyword was used as a query to find relevant posts. For the drug review websites and health Web forums, we built custom crawlers in Java using the jsoup [28] library for crawling and parsing the hypertext markup language (HTML) content. For each source, we collected the available data, including user information, posts, disorder, or condition under which a discussion appears, keywords, tags, etc*.* We emphasize that we collected only publicly available data in accordance with each site’s terms of use; no private data were collected.

### User Demographics Estimation Methods

#### Overview

We chose five demographic attributes as shown in Table 2: gender, age, ethnicity, location, and writing level. Since these attributes are not available in every source, we created several classifiers to derive missing attributes as specified in Table 2. Note that we do not fill missing values of users for sources that provide this information for at least some of their users, for example, if a user does not provide their age in Google+, we just ignore this user from the age-related analysis. Multimedia Appendix 1 shows the percentages of users who report each attribute in each source.

#### Gender

Four out of eight sources (Google+Health, DailyStrength/Treatments, WebMD/Drugs, and DailyStrength/Forums) allow users to report their gender (as shown in Table 2). Approximately 80% of the users of these sources chose to report it; thus, the reported gender was used for these sources.

For the other sources where gender is not available, we extended the methods of Mislove et al [13] to classify gender using the reported first name of users, if available; otherwise we extracted first names from user screen names. Note that screen names have not been used before, to the best of our knowledge, for gender estimation. In particular, we first collected the 1000 most popular male and female birth names reported by the US Social Security Administration [29] for each year from 1935 to 1995. Thus, we collected the names of people in 2014 aged 19-79 years old, which constitutes about 73.9% of the population [30]. There are 55,973 unique names in total. We further filtered this list to remove names with an aggregated frequency less than 10,000 or a discriminative gender probability less than 95%. The resultant list contained 1328 names. For TwitterHealth and Google+Health, we checked if one of these 1328 first names is contained in the user-specified name to classify the user’s gender. We first cleaned the first name by removing non-alphabetical characters and then performed case-insensitive string matching. Gender classifier evaluation is reported in Multimedia Appendix 1; the accuracy ranges from 76% to 99%.

#### Age

Similarly, age was also reported in four sources (Google+Health, DailyStrength/Treatments, WebMD/Drugs, and DailyStrength/Forums).Three sources display the age as a single number, whereas one source displays age as a range (eg, 35-45). Approximately 61% of the users of these sources reported their age. When users provide an age range, the total number of users for each range is distributed uniformly to each year in the range. Ages are then grouped into five age groups: 0-17, 18-34, 35-44, 45-64, and 65 years and older. These age ranges are also used by the US census [31].

#### Ethnicity

The ethnicity of the users is not reported in any of the sources that we study; therefore, we created an ethnicity classifier similar to Mislove et al [13]. The 2000 US census, which is the most recent available, reports the distribution of ethnicities for each last name (last names with less than 100 individuals were omitted) [32]. For example, the distribution for Hernandez is reported as 4.55% white, 0.38% black, 0.27% Asian, and 93.81% Hispanic. We filtered this list to remove the last names with a frequency less than 1000, or where the discriminative probability of the majority ethnicity is less than 80%. We then use the ethnicity with the majority probability to classify ethnicity based on last name for sources that include the last name of users (Google+Health and TwitterHealth). We understand that race and ethnicity are not the same especially when referring to Hispanics, but in this paper we try to simplify the presentation by only reporting ethnicity, that is, we do not distinguish groups like white Hispanic versus black Hispanic, but only Hispanic. For the other sources (health Web forums and drug review websites), which do not have user names, we found that using the screen name for ethnicity estimation is inaccurate, and hence we do not report on the ethnicity of these sources. Ethnicity labeling and classifier evaluation is reported in Multimedia Appendix 1.

#### Writing Level

Different methods and formulas for measuring readability are available using different factors such as average number of syllables per words, average number of words per sentences, or average number of letters per words. In our work, we used the Flesch-Kincaid Grade Level [16] formula to estimate the writing level (values generally correspond to school grades 1-12) of the users:

Flesch-Kincaid Reading Age = (0.39 x ASL) + (11.8 x ASW) – 15.59, where ASL is the Average Sentence Length, and ASW is the Average number of Syllables per Word.

Note that since we can only observe the text authored by users, we measure the writing level and not the reading level; however, we use the reading level formula since no alternative formula for the writing level exists. The writing level of a user is computed using the above equation by concatenating all of the user’s posts and personal description. Links and hashtags from tweets are removed, and users with less than 100 words in total are ignored. We found that very high reading level was being assigned to users who write very long incomprehensible sentences. This is a case that was not considered by the original Flesch-Kincaid reading age formula, which assumed that the text is grammatically and syntactically correct (eg, the text of a novel). For that, we omit sentences with more than 30 words.

## Results

### Overview

To put our results in perspective, we compare them with other general demographics statistics. The population and Internet usage for each demographic group was obtained from the US census [29,30], while other statistics for Twitter and Google+ came from other sources [11,33-35]. Further, we compare the demographics of the users participating in health-related discussions on Twitter and Google+ to the overall demographics of the users in these sites. All our results are statistically significant, except the comparison between health Web forums and drug review websites with respect to gender and age group (0-17). Also, there is no significant difference between Google+Health and drug review websites for age group (35-44).

### Gender

As shown in Table 3 [11,30,31,33], the gender distribution in the population and Internet usage is almost the same, and there is a slight difference for general social networks. Our first key finding is that drug review websites and health Web forums are dominated by female users; the number of female users is almost four times larger than that of male users. TwitterHealth and Google+Health have similar gender ratios when compared to the overall user base of Twitter and Google+.

The finding that women use health forums much more than men is partially supported by previous research, which shows that women report ill health more frequently than men [36]. In contrast, this is not true for Twitter and Google+, which are dominated by news exchanges [37].

**Table 3:** Gender distribution for TwitterHealth, Google+Health, drug reviews, health forums, compared to other relevant populations.

Source	Females, %	Males, %
Population [30]	51.05	48.95
Internet Use [31]	51.63	48.37
General social networks [11]	54.68	45.32
Twitter [33]	57.00	43.00
Google+ [33]	37.00	63.00
TwitterHealth^a^	51.81	48.19
Google+Health^a^	35.36	64.64
Drug review websites^a^	78.48	21.52
Health Web forums^a^	78.41	21.59

^a^These results are from this work. Results in the rows above are reported in the respective citations.

### Age

Table 4 [30,31,34,38] reports the age distribution of users in the studied social outlets and in other relevant sources, to put the results in perspective. Age groups were chosen based on the US census. We therefore understand that the age ranges are not equal, but since our main goal is comparing the demographics of Web-based health-related social outlets to other statistics such as Internet usage, we chose to follow the census age ranges in computer and Internet access. Further, we provide population distribution in the Table 4 to compare each group size with others. One-fifth of Internet users are in the group 0-17; this percentage drops to approximately 1% for drug review websites and health Web forums. The majority of users on drug review websites are between 45 and 64 years old, and drug reviews have more users over 65 years than any other source; this is expected as older patients use more medications [39]. However, the percentage of drug review users above 65 is slightly lower than the percentage of Internet users over 65, which means that older people still have low participation in Health 2.0 sites. Also, the 18-34 age group dominates health Web forums, which is congruent with general social networks usage [34]. To summarize, our second key finding is that the participants of health-related social outlets are generally older than those of general-purpose social forums, but still relatively low in the 65+ bracket. This is expected to change in the near future based on the participation statistics in the 45-64 bracket.

**Table 4:** Age distribution for Google+Health, drug reviews, health forums, and other relevant populations.

Source	0-17 years, %	18-34 years, %	35-44 years, %	45-64 years, %	65+ years, %
Population [30]	24.00	23.11	12.93	26.53	13.44
Internet use [31]	19.30	27.55	14.99	28.36	9.80
General social networks [34]	14.58	27.43	20.68	30.98	6.32
Google+ [38]	8.08	71.61	11.08	7.82	1.42
Google+Health^a^	3.42	53.21	21.89	19.02	2.46
Drug review websites^a^	1.05	31.13	22.36	36.84	8.62
Health Web forums^a^	1.03	39.80	25.81	28.95	4.41

^a^These results are from this work. Results in the rows above are reported in the respective citations.

### Ethnicity

For the ethnicity and location analyses, we focus on the US population, in order to compare to available US census statistics. Table 5 [12,31,40,41] shows the results of our ethnicity analysis. Recall that users’ ethnicity in Google+Health and TwitterHealth is classified using our last name-based classifier. Our third key observation is that blacks are underrepresented in health-related social network discussions (Google+Health, TwitterHealth).

**Table 5:** Ethnicity distribution for TwitterHealth, Google+Health, and other relevant populations.

Source	Asian, %	Black, %	Hispanic, %	White, %
Population [40]	4.5	12.2	15.8	65.1
Internet use [31]	5.5	11.7	13.9	67.2
General social networks [12]	5.3	12.1	14.5	66.5
Twitter [41]	N/A	9	12	71
TwitterHealth^a^	3.24	0.3	23.5	73.0
Google+Health^a^	5.6	0.3	17.4	76.6

^a^These results are from this work. Results in the rows above are reported in the respective citations.

### Location

Location is reported in four sources: the two general social networks (TwitterHealth, Google+Health), one drug review website (DailyStrength/Treatments), and one health Web forum (DailyStrength/Forums); approximately 62% of users reported their locations. For TwitterHealth and Google+Health, users report their location using a single string (eg, “NY, NY”). Thus, these strings are further processed to obtain structured locations (eg, state: New York, city: New York). In particular, non-alphanumeric characters and extra spaces were removed, and location strings with a frequency less than 14 were removed. This left us with about 60% of TwitterHealth and Google+Health users with location strings. Each location string was mapped to a location (city, state, country) using the Google Geocoding API [27]. We focus on US users, and hence we remove users from other countries. DailyStrength/Treatments and DailyStrength/Forums list the user’s city and state separately; thus, we use the reported state for these sources.

**Figure 2:**
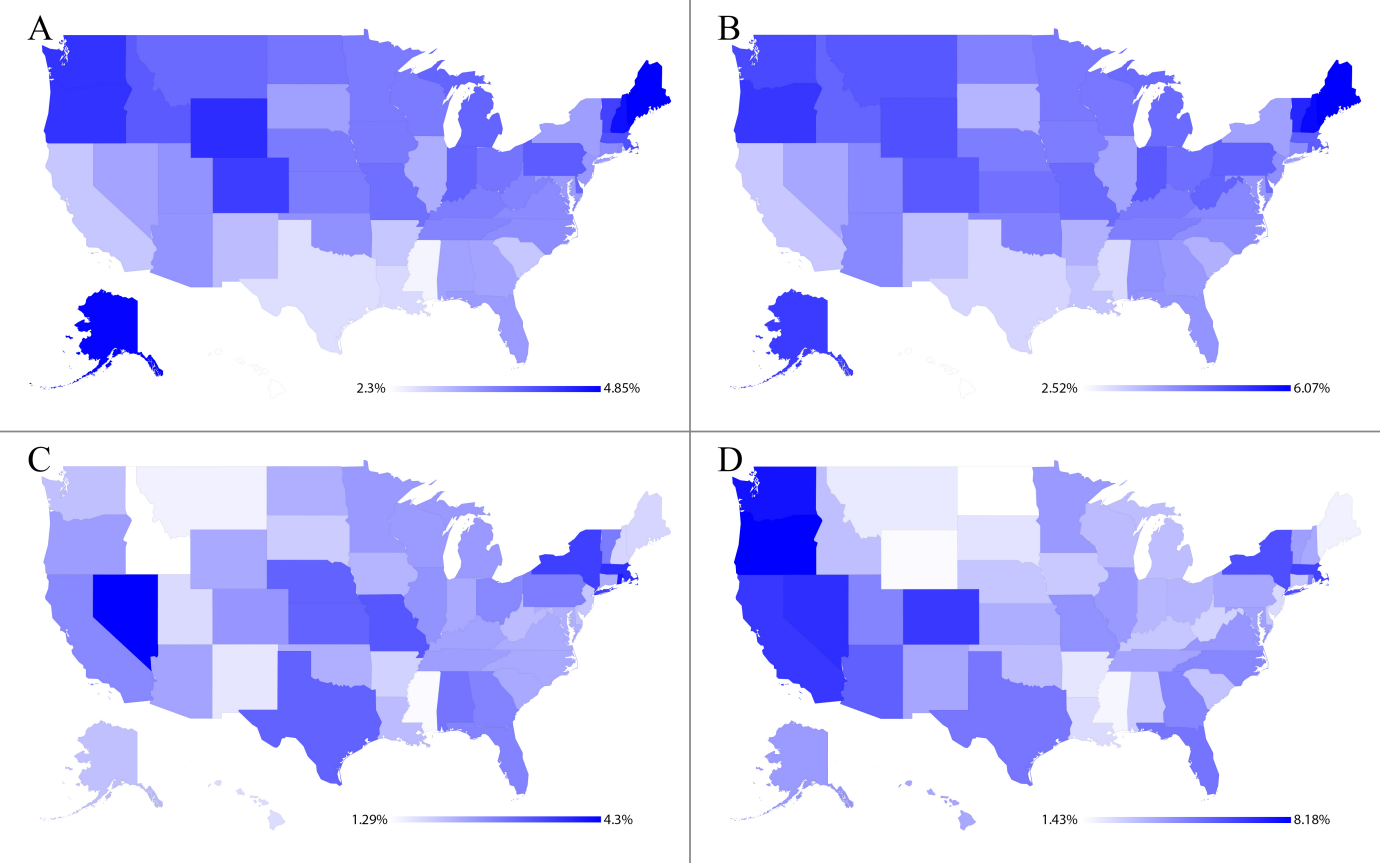
Per state capita number of users in (A) health web forums, (B) drug review websites, (C) TwitterHealth, and (D) Google+Health.

In Figure 2, we show the distribution of users for each type of Web-based health-related social outlet, normalized by state population. Panel A shows the distribution of users in health Web forums, Panel B shows the distribution of users in drug reviews websites, Panel C shows the distribution of users in TwitterHealth, and Panel D shows the distribution of users in Google+Health combined.

To better understand these results, we created Table 6 [31,42-45], which shows the correlation across all states between the normalized (by population) number of users in various health-related social outlets and other societal measures (see Multimedia Appendix 1 for more details). Our fourth key finding is that users in areas with higher income and more access to health care are more likely to participate in Web-based health-related outlets, and particularly in Web forums and drug review sites, which are the primary social sites for health-related information sharing [10]. Further, we see that in Twitter and Google+ the correlation with the number of physicians and education is higher. A reason could be that 59.1% of the 878,194 US active physicians [42] participate in these networks [46], which is a significant number, as the geolocated subsets of the Google+Health and TwitterHealth datasets contain only 882,207 users in the United States. The high correlation with education may be explained by the high percentage (91%) of Twitter users with college degree or higher [47].

**Table 6:** Correlation across all states between the normalized (per capita) number of users for each type of health-related social outlets, and each state’s population, normalized number of Internet users, normalized number of physicians, normalized number of uninsured patients, average annual income, and percentage of population with college degree or higher.

Correlation	Health Web forums	Drug review websites	TwitterHealth	Google+Health	Google+
Internet usage [31]	0.19	0.28	0.01	-0.01	0.00
No. of physician [42]	0.37	0.19	0.88	0.80	0.44
Uninsured population [43]	-0.40	-0.40	-0.17	-0.11	-0.10
Annual income [44]	0.38	0.27	0.17	0.25	0.26
Education (ratio of people with a college degree) [45]	0.35	0.22	0.56	0.63	0.54

### Writing Level

The writing level, as previously mentioned, is measured using a standard reading level formula that assigns a school grade to the given text. For example, when a person writes text at a 5^th^ grade reading level, it implies that their writing should be understood by people that have passed the 5^th^ grade. Table 7 reports our results for writing level of health-related social outlet users. We see that Google+Health users have generally higher writing level than the rest sources, which may mean that more of the Google+Health users are professional accounts.

**Figure 3:**
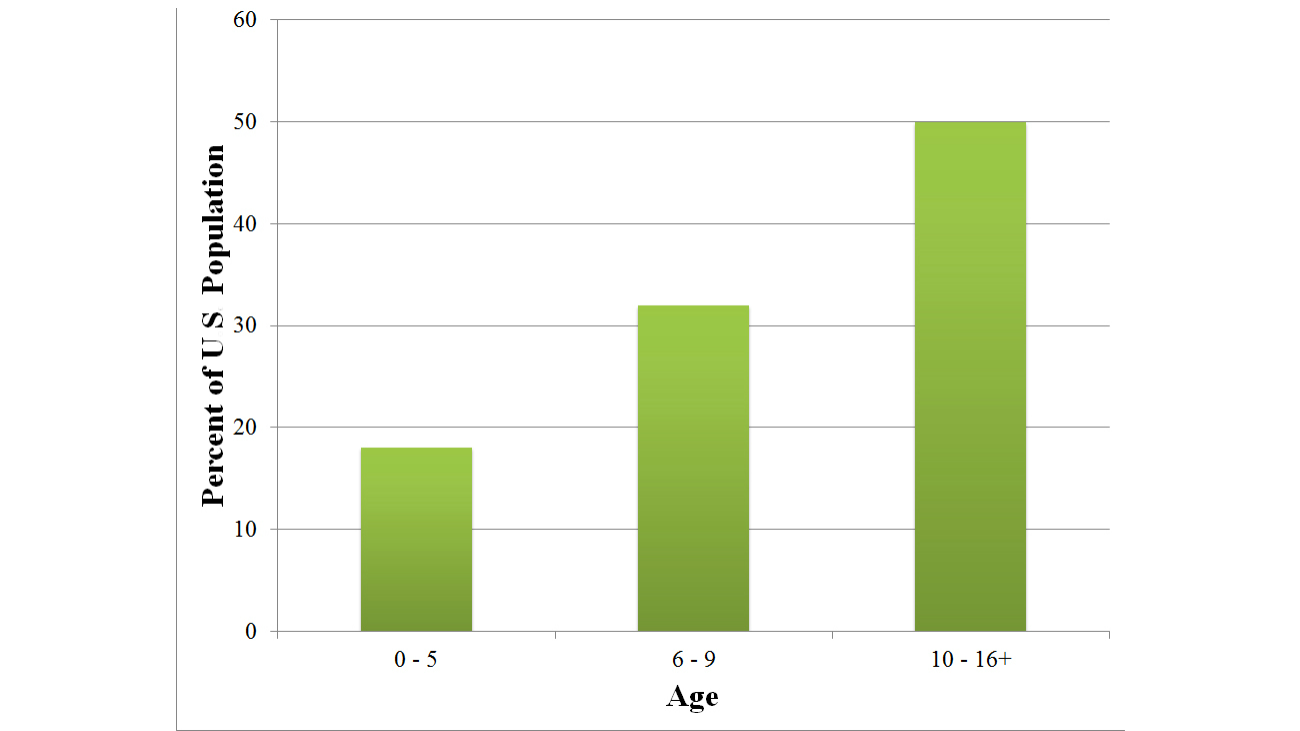
Reading level of US population.

Next, we try to put these findings in perspective. Unfortunately, related work reports only on reading levels (and not writing levels) of the US population participating in social outlets. Thus, we compare our results in Table 7 to Figure 3, which reports the reading level of the general US population [15].

**Table 7:** Writing level distribution for TwitterHealth, Google+Health, drug reviews, and health forums.

Source	Age 0-5, %	Age 6-9, %	Age 10-16, %
TwitterHealth	37.77	51.09	11.13
Google+Health	6.45	55.63	37.91
Drug review websites	30.42	66.17	3.41
Health Web forums	28.79	68.24	2.98

Our fifth key finding is that the writing level in health-related social outlets (Table 7) is generally lower than the reading level of the population (Figure 3). Thus users/patients can easily comprehend the posts and hence benefit from the experiences of other users. The benefit of social interaction with respect to health empowerment has been demonstrated before [48]. In an online epilepsy community, 59% of patients found another patient experiencing the same symptoms, 58% had a better understanding of seizures, and 55% learned more about treatments and symptoms.

### Statistical Significance Tests

Tables 8 and 9 report the *P* values for Pearson’s chi-square test of independence and the Mann-Whitney U test. Note that we compute only significance values between sources that we have analyzed and not between our sources and sources analyzed by other works (such as Google+ [38]), since we do not have the raw data for those sources.

**Table 8:** *P* values for Pearson’s chi-square test of independence.

	Gender	Age	Ethnicity	Writing level
TwitterHealth vs Google+Health	<.001	N/A	<.001	<.001
TwitterHealth vs Health Web forums	<.001	N/A	<.001	<.001
TwitterHealth vs Drug review websites	<.001	N/A	<.001	<.001
Google+Health vs Health Web forums	<.001	<.001	<.001	<.001
Google+Health vs Drug review websites	<.001	<.001	<.001	<.001
Health Web forums vs Drug review websites	<.001	<.001	<.001	<.001

**Table 9:** *P* values for Mann-Whitney U test.

		TwitterHealth vs Google+Health	TwitterHealth vs Health Web forums	TwitterHealth vs Drug review websites	Google+Health vs Health Web forums	Google+Health vs Drug review websites	Health Web forums vs Drug review websites
**Gender**							
	Male	<.001	<.001	<.001	<.001	<.001	.5797
	Female	<.001	<.001	<.001	<.001	<.001	.5797
**Age**							
	0-17	N/A	N/A	N/A	<.001	<.001	.5144
	18-34	N/A	N/A	N/A	<.001	<.001	<.001
	35-44	N/A	N/A	N/A	.01661	.7747	<.001
	45-64	N/A	N/A	N/A	<.001	<.001	<.001
	≥65	N/A	N/A	N/A	.01066	<.001	<.001
**Ethnicity**							
	White	<.001	<.001	<.001	<.001	<.001	.1316
	Black	.6339	<.001	<.001	<.001	<.001	.0944
	Asian	<.001	<.001	<.01	<.001	<.001	.8054
	Hispanic	<.001	<.001	<.001	<.001	<.001	.6503
**Writing level**							
	0-5	<.001	<.001	<.001	<.001	<.001	<.001
	6-9	<.001	<.001	<.001	<.001	<.001	<.001
	10-16	<.001	<.001	<.001	<.001	<.001	.00516

## Discussion

### Principal Findings

Our results can help health care providers customize educational campaigns for different groups. For example, white women should be informed to a larger extent on the possible misinformation spreading in health Web forums, since they participate much more.

Regarding mitigating ethnicity-based health care disparities, we found that Twitter and Google+ are more effective in reaching out to Hispanics about health care offerings. However, this is not true for black ethnicity, who are not overrepresented in any health-related social outlet. This means that there is no single outlet to reach black population, which has been shown to receive worse health care by about 40% comparing to white population [49].

Advertisers may use our results to decide on the best sites to advertise their products; for instance, drug review websites are more appropriate than Google+ to advertise drugs for the 45-64 age bracket, but the opposite is true for the 18-34 age bracket. Further, drug review websites and health Web forums are better to target females when advertising for their products than other health-related social outlets.

In the age results section, we found that younger groups (18-34 years old) participate in large numbers in health forums, which may sound counterintuitive. By analyzing posts for this age bracket, we found the most popular keywords are related to pregnancy such as birth control, ovulation, and miscarriage. On the other hand, their participation is lower for drug review websites. A possible explanation may be that often patients who talk about pregnancy are not taking any drugs, compared to other conditions like diabetes, where drugs are more common.

We also attempt to explain the disparities in the participation in health-related social outlets based on socioeconomic factors through the state-level participation distributions. Our results in Table 6 show that less access to physicians does not lead to higher participation in health-related social outlets as one would expect. In contrast, it seems that the participation in such outlets is correlated with the access to health care and the average income.

The weak but positive correlation between income and participation to health Web forums and drug review sites may be partially attributed to the higher Internet usage of the more affluent groups, as shown in Table 6. Another possible explanation is that lower income or uninsured persons are more likely to be part of a community with health care disparities [50].The positive correlation between education and participation in health-related social outlets, especially Google+Health and TwitterHealth, may be partially explained by the fact that people with college degrees are less likely to be uninsured, since 10% of college graduates are uninsured, compared to 40% of adults who have not graduated from high school [51]. In addition, 60% of uninsured people are from families with low incomes [51], and the group of people with income lower than US 30K is the lowest group in terms of accessing health information [52], Hence, our results show that people with low income have less access to health information.

On the other hand, we found that the content in health-related social outlets is easy to understand for almost all users, given the low writing level. That is, the well-known health literacy issue, which is more severe in low-income and lower education populations [5], does not seem to apply to Web-based health-related social outlets. Of course, the low writing level does not address the issue of language, as many low income and low education users in the United States do not speak English at home [53].

### Limitations

Our ethnicity and gender classifiers are not perfect, as shown in Multimedia Appendix 1, and thus introduce an error into our analyses. This issue is less significant for gender, since out of all users included in our gender analysis for health Web forums and drug review websites, a majority of the users (over 94%) report their gender, and hence the classifier was only used for 6% of users. Further, a majority of users in drug review websites and health Web forums are female, and our gender classifier obtained an accuracy greater than 99% for females when using a screen name.

Another limitation is the informal writing style of social media posts, as our writing level method uses the average sentence length, which expects that posts are properly punctuated. We addressed this limitation to some degree by only considering sentences of a reasonable length (less than 30 words). Estimating writing level could have been improved by considering other features like typos or spelling mistakes. Further, it would be useful to measure the quality of the posted information, in addition to just the writing level. This is a challenging issue, which we leave as future work.

Since all the attributes are reported by users, there is inevitably self-selection bias. In particular, gender, age, and location are not mandatory in any site. For instance, older people may choose not to report their age. Moreover, choosing to report the real names or posting profile pictures could also create self-selection bias in our gender and ethnicity classifiers. There may also be various types or degrees of bias across different outlets. For instance, WebMD users may use their real name less frequently than Twitter users. This in turn may bias the study results, especially for ethnicity where we depend completely on the classifier results.

### Conclusion

We studied user demographics in Web-based health-related social outlets, which we split into three different types: social networks, drug review websites, and health Web forums. The distributions of the demographic attributes—gender, age, ethnicity, location, and writing level—have been analyzed for each source type and compared with relevant baseline user distributions like Internet and general social outlets participation. The results reveal interesting and often unexpected disparities with respect to all demographic attributes.

## Appendix

Multimedia Appendix 1 Online social outlets summary, health keywords, classifiers evaluation, and data coverage.

## Abbreviations

API: application programming interface
