# Supplementary material for: A Study of the Demographics of Web-Based Health-Related Social Media Users
Source: J Med Internet Res. 2015 Aug 6;17(8):e194. doi: 10.2196/jmir.4308 (PMC4705027; doi:10.2196/jmir.4308)
Supplement: Multimedia Appendix 1 [file jmir_v17i8e194_app1.pdf]

Appendix

A: Online Social Outlets Summary

Table A.1 summarizes the used sources in our work, including the web address, and the start and end dates for collected posts. For TwitterHealth, we use a sample of 10%.

Table A.1 The start and end dates for sources represent the time of first post and last post. Not Applicable (N/A) is used for sources that do not log each post's creation date.

Dataset	URL	Start	End
TwitterHealth [1]	www.twitter.com	May 1, 2013	Nov. 15, 2013
Google+Health [2]	plus.google.com	Aug. 24, 2009	Jan. 05, 2014
Drugs.com [3]	www.drugs.com	Feb. 16, 2007	Jan. 26, 2014
DailyStrength / Treatments [4]	www.dailystrength.org/treatments	N/A	N/A
WebMD / Drugs [5]	www.webmd.com/drugs	Sep. 17, 2007	Nov. 27, 2013
Drugs.com / Answers [6]	www.drugs.com/answers	Mar. 25, 2004	Feb. 02, 2014
WebMD [7]	www.webmd.com	Dec. 31, 1999	Feb. 07, 2014
DailyStrength/Forums [8]	www.dailystrength.org/support-groups	Jun. 21, 2006	Jan. 25, 2014

B: Health Keywords

The list of used keywords to filter health-related posts from Twitter and Google+:

Table B.1 Used keywords to filter health-related posts from Twitter and Google+

Drugs

Abilify	Clonidine	Glyburide	Naproxen	Suboxone
Actonel	Combivent	Hydrochlorothiazide	Nasonex	Sulfamethoxazole
Actos	Concerta	Hydrocodone	Nexium	Synthroid
Advair	Crestor	Ibuprofen	Niaspan	Toprol
Albuterol	Cyclobenzaprin	Isosorbide	Nuvaring	Tramadol
Alendronate	Cymbalta	Januvia	Omeprazole	Trazodone
Allopurinol	Detrol	Klor-Con	Oxycodone	Triamterene
Alprazolam	Diazepam	Lantus	Oxycontin	Tricor
Ambien	Digoxin	Levaquin	Pantoprazole	TriNessa
Amlodipine	Diltiazem	Levothyroxine	Paroxetine	Ventolin
Amoxicillin	Diovan	Levoxyl	Penicillin	Verapamil
Amphetamine	Doxycycline	Lexapro	Plavix	Viagra
Aricept	Effexor	Lipitor	Potassium	Vitamin
Atenolol	Enalapril	Lisinopril	Pravastatin	Vytorin
Azithromycin	Famotidine	Loestrin	Premarin	Vyvanse
Benazepril	Fexofenadine	Lorazepam	Proair	Warfarin
Benicar	Flomax	Lovastatin	Promethazine	Warfarin
Carisoprodol	Flovent	Lovaza	Propoxyphen	Xalatan
Carvedilol	Fluconazole	Lyrica	Proventil	Zetia

Cefdinir	Fluoxetine	Meloxicam	Ranitidine	Zolpidem
Celebrex	Fluticasone	Metformin	Seroquel	Zyprexa
Cephalexin	Folic	Methylprednisolone	Sertraline	Amitriptyline
Cialis	Furosemide	Metoprolol	Simvastatin	Cheratussin
Ciprofloxacin	Gabapentin	Mupirocin	Singulair	Ocella
Citalopram	Gabapentin	Namenda	Spiriva	Prednisone
Clonazepam				
		**Hashtags**		
#BCSM	#HCSM	#hcsmeu	#HITsm	#mhsm
#doctors20	#hcsmca	#hcsmin	#Ideagoras	#RareDisease
#eldercarechat				
		**Disorders**		
AIDS	Constipation	Heart disease	Liver disease	Otitis
Alzheimer	COPD	Hemochromatosis	Lupus	Overweight
Anxiety disorders	Crohn's disease	Hepatitis	Lyme disease	Parkinson's
Arthritis	Cystic fibrosis	Herpes	Lymphoma	Pelvic inflammatory disease
Asthma	Dementia	High cholesterol	Meningitis	Pertussis
Astigmatism	Depression	HIV	Meningococcal	Prostate disorder
Autoimmune	Diabetes	Hodgkin's disease	Menopause	Raynaud's Phenomenon
Bipolar	Eczema	HPV	Mental illness	SARS
Cancer	Endometriosis	Hypertension	Migraine	Sexually transmitted disease
Candidiasis	Fibroids	Impotence	Multiple sclerosis	
Cataracts	Fibromyalgia	Insomnia	Muscular dystrophy	
Celiac	Flu	Irritable bowel		

Chicken pox<br>Chlamydia<br>Chronic fatigue syndrome<br>Cold sore<br>Common cold	Food poisoning<br>Gallstones<br>Gonorrhea<br>Grave's disease<br>Hay fever<br>Headache	syndrome<br>Jaundice<br>Kidney disease<br>Lactose intolerance<br>Leukemia	Myopia<br>Narcolepsy<br>Non-Hodgkin's lymphoma<br>Obesity<br>Osteoporosis	Sleep disorder<br>Stroke<br>Thrush<br>Thyroid<br>Whooping cough
		**Pharmaceuticals**		
Johnson & Johnson<br>Pfizer<br>Roche	GlaxoSmithKline<br>Novartis<br>Sanofi	AstraZeneca<br>Abbott	Merck<br>Bayer	Eli Lilly<br>Bristol-Myers
		**Insurance**		
healthcare<br>health insurance<br>medicare<br>medicaid<br>AARP<br>Aetna<br>Aflac<br>American Family Insurance<br>American Fidelity Assurance<br>American National	Company<br>Amerigroup<br>Anthem Blue Cross<br>Assurant<br>Bankers Life and Casualty<br>Blue Cross and Blue Shield<br>Centene<br>Cigna<br>Conseco	Coventry Health<br>EmblemHealth<br>Fortis<br>Golden Rule Insurance<br>Group Health Cooperative<br>HealthNet<br>HealthMarkets<br>HealthSpring<br>Highmark Insurance	Humana<br>Independence Blue Cross<br>Kaiser Permanente<br>Kaleida Health<br>LifeWise Health Plan of Oregon<br>Medical Mutual of Ohio<br>Molina Healthcare<br>Mutual of Omaha<br>Premera Blue	Principal Financial Group<br>The Regence Group<br>Tricare<br>Shelter Insurance<br>Thrivent Financial<br>UnitedHealth<br>Unitrin<br>Universal American Corporation

Insurance	Cross	WellCare Health<br>WellPoint

C: Classifiers Evaluation

C.1 Gender classifiers evaluation

We evaluated our method for classifying gender using Google+Health and Health OSNs users where users reported their gender; Health OSNs where users reported their gender include DailyStrength/Treatments, DailyStrength/Forums, and WebMD / Drugs. Google+Health and the three Health OSNs respectively have 44,614 users and 25,603 users that reported their gender. The resulting confusion matrixes are shown in tables C.1 and C.2 for Google+Health and the three Health OSNs, which achieved accuracies of 98.74% and 76.29% respectively. As we expected, our classification using first name is more accurate than using screen name; nevertheless, our screen name accuracy is reasonable, and much higher for females (greater than 99%). Thus, classification errors using screen name is further reduced due to the fact that the number of females in drug reviews and health forums is much larger than the number of males.

Table C.1 Confusion matrix between the reported genders on Google+Health and our gender classifier using first name.

	**Classified female**	**Classified male**
Female	15328	226
Male	334	28726

Table C.2 Confusion matrix between the reported genders on three Health OSNs and our gender classifier using screen name.

	Classified female	Classified male
Female	18504	1028
Male	177	5894

C.2 Ethnicity classifiers evaluation

In order to evaluate the ethnicity classifier for Google+Health and TwitterHealth, we chose from Google+Health (we expect these results to carry to TwitterHealth) 50 users from each of the ‘White’, ‘Black’, ‘Asian’ and ‘Hispanic’ races that were identifiable by our classifier. Three authors (S.S., M.S., and M.W.) labeled these users using their profile picture. We only consider the subset of users for which at least two of the labelers agreed on, that is, we label based on majority vote. This left us with 128 labeled users to evaluate the classifier’s accuracy. The classifier’s accuracy is 81.25%, and the agreement measure for labelers is illustrated in table C.4. The following confusion matrix summarizes the results of applying the classifier to 128 users’ last names.

Table C.3 Confusion matrix between the labeled ethnicity on Google+Health and our ethnicity classifier using surename.

	Labeled based on profile picture			
**Classifier**	**Asian**	**Hispanic**	**Black**	**White**
Asian	22	0	0	1
Hispanic	2	30	0	10
Black	3	1	21	5
White	0	0	2	31

Table C.4 Agreement measure (Cohen's kappa) between three labelers for Google+Health, Health Web Forums and Drug Review Websites

**Labelers**	**Google+Health (based on image and last name)**
M.S, M.W	0.7
M.S, S.S	0.78
M.W, S.S	0.78

D: Data Coverage

D.1 Percentages of reported attributes for each source

Table D.1 shows the percentages of each reported attributes for each source. As indicated in the table's caption, the percentage might be reported by the users or calculated by our classifiers. For gender, we only consider users whose reported first names or screen names are matched to the list of popular names we extracted from U.S. Social Security Administration (as described in the method section). Similarly, for ethnicity classifier we match reported last names with U.S. Census last name list to find the ethnicity with the highest distribution; however, here we don't consider reported screen names, because they led to lower accuracy, and hence we don't include them in the results. We see that only two sources have last names and hence we report no ethnicity information for the rest. For writing level classifier, as mentioned in the methods, we only consider users who have total length of combined posts more than 100 words. Since each Twitter post has maximum length of 140 characters, and we remove links and hashtags, the percentage of users who have concatenated posts length longer than 100 words is small. The N/A entries in the tables are either not reported by the source, or the features needed by the classifiers are not available.

Table D.1 The percentage of reported attribute for each source. Percentages with (*) indicate that the attributes were calculated by our classifiers. Percentages with (**) indicate reported locations in the United States

Source	Gender	Age	Ethnicity	Location	Writing Level
TwitterHealth	10.49% *	N/A	10.54% *	16.99% **	2.49% *
Google+Health	66.29%	1.13%	16.4% *	17.55% **	15.89% *
DailyStrength/Treatment	82.46%	73.25%	N/A	58.47% **	53.68% *
Drugs.com	4.26% *	N/A	N/A	N/A	15.99% *
WebMD / Drugs	96.47%	96.08%	N/A	N/A	13.54% *
DailyStrength/Forums	74.93%	65.23%	N/A	51.66% **	81.7% *
Drugs.com / Answers	6.51% *	N/A	N/A	N/A	11.14% *
Webmd	6.25% *	N/A	N/A	N/A	50.96% *

D.2 Users distribution among states

Table D.2 shows the percentages of users in each state that participate to the social outlets considered in this paper, that is, we divide the number of users in each state to the population of that state. These distributions were compared with the number of physician, uninsured population, and ratio of people with college degree.

Table D.2 Distribution of users among states

**State**	***Health Web Forums***	***Drug Review Websites***	***TwitterHealth + Google+Health***	**No. of Physicians per 100,000** [9]	**Uninsured population** [10]	**Ratio of people with college degree** [11]
AL	3.21%	4.01%	0.30%	321	14.44%	22
AK	4.63%	5.06%	0.21%	481	18.44%	26.6
AZ	3.37%	4.17%	0.24%	368	18.16%	25.6
AR	2.83%	3.56%	0.18%	301	18.10%	18.9

CA	2.84%	3.25%	0.28%	351	18.99%	29.9
CO	4.14%	4.72%	0.26%	354	14.06%	35.9
CT	3.39%	4.04%	0.23%	471	9.30%	35.6
DE	3.76%	4.46%	0.21%	528	10.69%	28.7
DC	4.54%	4.63%	2.33%	1576	9.69%	48.5
FL	3.26%	3.94%	0.28%	336	20.65%	25.3
GA	3.13%	3.86%	0.28%	320	19.29%	27.5
HI	2.21%	2.42%	0.17%	623	7.78%	29.6
ID	3.89%	4.61%	0.13%	321	17.26%	23.9
IL	3.09%	3.77%	0.26%	334	14.40%	30.6
IN	3.76%	4.71%	0.23%	406	12.92%	22.5
IA	3.55%	4.24%	0.22%	364	10.80%	25.1
KS	3.51%	4.37%	0.32%	379	12.94%	29.5
KY	3.55%	4.31%	0.24%	380	14.96%	21
LA	2.60%	3.25%	0.21%	359	19.67%	21.4
ME	4.82%	6.03%	0.18%	466	9.61%	26.9
MD	3.28%	3.94%	0.23%	486	13.01%	35.7
MA	4.00%	4.69%	0.38%	508	4.35%	38.2
MI	3.79%	4.52%	0.25%	453	12.10%	24.6
MN	3.56%	4.31%	0.25%	375	9.06%	31.5
MS	2.36%	2.99%	0.14%	320	17.50%	19.6
MO	3.68%	4.54%	0.33%	420	14.04%	25.2
MT	3.73%	4.77%	0.15%	415	18.20%	27.4
NE	3.56%	4.26%	0.32%	464	12.97%	27.4
NV	3.16%	3.68%	0.44%	276	22.50%	21.8
NH	4.65%	5.88%	0.18%	472	11.59%	32
NJ	3.28%	4.01%	0.20%	397	15.02%	34.5
NM	2.91%	3.35%	0.16%	408	20.96%	25.3
NY	3.22%	3.78%	0.36%	432	12.85%	32.4
NC	3.36%	3.99%	0.24%	341	16.87%	26.5
ND	3.54%	4.10%	0.22%	497	11.28%	25.8
OH	3.63%	4.45%	0.27%	361	13.19%	24.1
OK	3.33%	4.17%	0.23%	325	17.14%	22.7
OR	4.27%	5.23%	0.25%	359	15.07%	29.2
PA	3.89%	4.69%	0.29%	425	11.24%	26.4
RI	3.47%	4.74%	0.40%	410	11.93%	30.5
SC	2.81%	3.54%	0.23%	314	17.89%	24.3
SD	3.17%	3.47%	0.19%	435	13.55%	25.1
TN	3.48%	4.25%	0.25%	331	13.91%	23
TX	2.59%	3.06%	0.32%	264	24.33%	25.5

UT	3.33%	4.10%	0.18%	317	14.26%	28.5
VT	4.15%	5.50%	0.29%	547	8.24%	33.1
VA	3.36%	4.07%	0.23%	390	13.28%	34
WA	4.28%	5.00%	0.21%	375	13.97%	31
WV	3.47%	4.60%	0.21%	380	14.30%	17.3
WI	3.58%	4.36%	0.25%	410	9.83%	25.7
WY	4.32%	4.86%	0.23%	514	16.84%	23.8

References

1. Twitter. https://twitter.com/.

2. Google+. https://plus.google.com/.

3. Drugs.com | Prescription Drug Information, Interactions & Side Effects. http://www.drugs.com/. Archived at: http://www.webcitation.org/6W23lHwIt

4. Treatments: reviews of drugs, therapies and remedies by everyday people – DailyStrength. http://www.dailystrength.org/treatments. Archived at: http://www.webcitation.org/6W23nXoJa

5. WebMD Drugs & Treatments - Medical Information and user ratings on prescription drugs and over-the-counter (OTC) medications. http://www.webmd.com/drugs/index-drugs.aspx. Archived at: http://www.webcitation.org/6W23rKUfB

6. Medical Questions Answered - Drugs.com. http://www.drugs.com/answers/. Archived at: http://www.webcitation.org/6W23vDAi5

7. WebMD - Better information. Better health. http://www.webmd.com/. Archived at: http://www.webcitation.org/6W247u0HQ

8. Online Support Groups - DailyStrength. http://www.dailystrength.org/support-groups. Archived at: http://www.webcitation.org/6W23ztpRe

9. Young A, Chaudhry HJ, Thomas J V, Dugan M. A Census of actively licensed physicians in the United States , 2012. J Med Regul 2012;99:11-24. https://www.fsmb.org/Media/Default/PDF/Census/census.pdf

10. Denavas-walt BC, Proctor BD, Smith JC. Income, poverty, and health insurance coverage in the United States: 2012, U.S. Census Bureau, Current Population Reports. Washington, DC; 2013:60-245. http://www.census.gov/prod/2013pubs/p60-245.pdf. Archived at: http://www.webcitation.org/6W2D1eglk

11. US Census Bureau. Statistical abstract of the United States: 2012; 2012:143-192. http://www.census.gov/prod/2011pubs/12statab/educ.pdf. Archived at: http://www.webcitation.org/6W2DHYzQJ
